# Effects of vaccines on clinical characteristics of convalescent adult patients infected with SARS-CoV-2 Omicron variant: A retrospective study

**DOI:** 10.3389/fmicb.2023.1096022

**Published:** 2023-03-30

**Authors:** Jingyu Wang, Henan Dong, Jie Zhao, Tianning Li, Meng Wang, Chunlei Zhou, Hong Mu

**Affiliations:** ^1^Department of Laboratory Medicine, Tianjin First Central Hospital, Tianjin, China; ^2^The First Central Clinical School, Tianjin Medical University, Tianjin, China

**Keywords:** SARS-CoV-2, COVID-19, Omicron variant, vaccine, clinical characteristics, adult patients

## Abstract

**Introduction:**

The protective effect of SARS-CoV-2 vaccines has become a global focus due to Omicron variant pandemic. The effects of various SARS-CoV-2 vaccines are diverse. However, studies on the effect of domestic vaccines on clinical characteristics in convalescent adult patients infected with the Omicron variant are lacking.

**Methods:**

In this retrospective, single-center cohort study, the effect of three domestic vaccines on clinical characteristics of convalescent adult patients infected with the Omicron variant was investigated in the initial largest outbreak of the Omicron variant infection between January and February 2022 in Tianjin, China. The primary endpoint was COVID-19 severity and the secondary endpoints were re-positive results on nucleic acid tests, liver and kidney function, and inflammation levels during recovery.

**Results:**

A total of 320 adult patients infected with the Omicron variant were enrolled, including 296 post-vaccination and 24 unvaccinated patients. The median age of the unvaccinated patients was higher than that of vaccinated patients, but no significant difference was detected in the sex composition ratio between the different groups. Binary logistic regression results suggested that Sinopharm and Sinovac vaccine was an independent protective factor for relieving the severity of the Omicron variant infection. Regrettably, the vaccines did not showed any protective effect on the liver and kidney function of convalescent adult patients. Three domestic vaccines significantly relieved inflammation and increased the SARS-CoV-2-specific antibody levels. Furthermore, Sinovac and CanSino vaccines had a better immune stimulation effect on increasing T lymphocytes levels in convalescent adult patients. In addition, three domestic vaccines have protective effects on preventing re-detectable positive (RP) result in convalescent adult patients.

**Conclusion:**

Although the three domestic vaccines cannot prevent the infection of the Omicron variant, it has a significant protective effect in adult patients. This study supports the policy of accelerating to vaccination worldwide combat the evolving and mutating SARS-CoV-2.

**Discussion:**

Omicron spreads faster and might escape antibodies more readily than previous variants, increasing the cases of reinfection and breakthrough infections in vaccinated people. Although vaccinated people are likely to have a much lower risk of severe disease from Omicron infection, many issues still need to be considered. Concerns about lower vaccine efficacy because of new variants might have changed our understanding of the COVID-19 endgame, disabusing the world of the notion that global vaccination is by itself adequate for controlling SARS-CoV-2 infection. The current data showed that vaccination with three domestic SARS-CoV-2 vaccines alleviates the disease severity of adult patients with COVID-19, reduces the inflammation level and the RP rate of convalescent adult patients, and enhances body’s defense against the virus in convalescent adult patients. Moreover, our study has highlighted that a combination prevention approach of vaccination and public health measures would be an effective strategy.

## 1. Introduction

The coronavirus disease 2019 (COVID-19) caused by the new coronavirus (SARS-CoV-2) is still breaking out and spreading on a large scale worldwide, causing serious damage to the global health care system and human thus health, and has gained global attention (Lu et al., 2020). As a respiratory transmitted virus, the transmission of SARS-CoV-2 has been associated with three primary modes known as “contact,” “droplet,” and “airborne” transmission (Priyanka et al., 2020). The worsening COVID-19 outbreak suggested other potential routes of transmission such as fecal-oral and aerosol transmission, which have been elucidated by various empirical and laboratory studies (Goh et al., 2013; Chen Y. et al., 2020; Tang et al., 2020; Xu et al., 2020; Heneghan et al., 2021), in addition to the three primary transmission modes. The adoption of social distancing, face masks, hand hygiene and environmental disinfection is considered an effective mitigation strategy, but does not prevent the outbreak of COVID-19 pandemic. Therefore, from the scientific community throughout the globe, there is an urgent and critical requirement of the research to interrupt the transmission and the infection of SARS-CoV-2.

SARS-CoV-2 is similar to other RNA viruses with a high mutation rate. As of 2022, multiple variants of SARS-CoV-2 have emerged and gradually become epidemic strains, including five variants of concern defined by the World Health Organization (WHO): Alpha, Beta, Gamma, Delta, and Omicron (Malik et al., 2022). Specifically, Omicron,^1^ recently discovered in Botswana, as the most mutated variant, spreads rapidly, is highly contagious, has a high viral load, and is likely to escape the host’s immune defense system, spreading widely around the world and becoming the dominant global epidemic strain, which has attracted widespread attention (Zhang X. et al., 2020; CDC COVID-19 Response Team, 2021; Harvey et al., 2021; Kannan et al., 2021; Schmidt et al., 2021; Rana et al., 2022; Tian et al., 2022). As of 6:08 p.m. CET, 14 October 2022, 621 million confirmed cases of COVID-19, including 6.5 million deaths, have been confirmed and reported to WHO.^2^

Currently, the Omicron variant has replaced the Delta variant as the most dominant epidemic strain worldwide.^3^ It not only poses new challenges to the prevention and control of COVID-19, but is also a huge threat to global human health. For COVID-19 pandemic, vaccination is still considered the most effective measure to prevent and combat the spread of SARS-CoV-2 (Štefan et al., 2021; Zhang et al., 2021). In order to block the spread of SARS-CoV-2, over the past 2 years, a large-scale SARS-CoV-2 vaccination program has been carried out globally. As of 11 October 2022, a total of 12,782,955,639 doses of SARS-CoV-2 vaccine have been administered globally (see text foot note 2), and >68.3% of the global population has received at least one dose of SARS-CoV-2 vaccine [Our World in Data. Coronavirus (COVID-19) vaccinations (EB/OL) (2022-03-25) (2022-03-26)].^4^ China began large-scale SARS-CoV-2 vaccination on December 15, 2020, and as of July 22, 2022, >3.4 billion doses have been administered nationwide; the number of vaccinated people with inactivated SARS-CoV-2 vaccine as the main type has reached 1,298,636,000 (Lai et al., 2022).^5^ Although a variety of vaccines have been utilized clinically, their effectiveness remains uneven, raising questions about their effectiveness and applicability.

Existing studies have shown that SARS-CoV-2 vaccines designed by different technical routes exert protective effects on preventing severe disease and reducing mortality (Lopez Bernal et al., 2021; McNamara et al., 2022). However, some studies have shown that antibody levels decrease several months after vaccination (Wang et al., 2021), thereby reducing the effectiveness of existing vaccines against SARS-CoV-2 (Chia et al., 2021; Sabino et al., 2021; Fiolet et al., 2022). In addition, as SARS-CoV-2 variants continue to emerge (He et al., 2022), the dominant virus strains of the global COVID-19 epidemic are constantly changing. On January 8, 2022, the first case of indigenous Omicron variant infection was confirmed in Tianjin (Sun et al., 2022), which subsequently spread across China.^6^ Nevertheless, the vaccine efficacy in preventing the infection was not satisfactory, and some vaccinated people were still infected with Omicron variant (Jamal et al., 2022; Lee et al., 2022; Zhang et al., 2022). Surprisingly, only a few studies have reported the disease severity of COVID-19, liver and kidney function indicators, inflammatory and immune indicators and RP rates in post-vaccination adults infected with Omicron. Therefore, clinical studies on post-vaccination patients infected with Omicron variant are essential to preventing the development of the epidemic. The present study aimed to comprehensively analyze the effect of the three domestic vaccines, including Sinopharm (BIBP-CorV vaccine), Sinovac (Corona vaccine), and CanSino vaccine (Ad5-nCoV vaccine), on the clinical features and laboratory test data of the adult patients infected with Omicron variant, in order to provide a reference and basis for vaccination and clinical treatment of adult populations.

## 2. Materials and methods

### 2.1. Study design

This was a retrospective and single-center cohort study. A total of 438 patients were diagnosed with SARS-COV-2 infection by real time-polymerase-chain-reaction (RT-PCR) assays in Tianjin Haihe Hospital (Tianjin, China) between January and February 2022. After discharge from Tianjin Haihe Hospital (Tianjin, China), the patients were transferred to the Tianjin First Central Hospital for 14 days of isolation, rehabilitation and medical observation. From January to March 2022, 320 convalescent adult patients infected with Omicron variant were admitted to Tianjin First Central Hospital and enrolled in this study. Daily follow-up in person and SARS-CoV-2 RNA detection were performed simultaneously. Diagnosis, clinical classifications, and complication definitions for COVID-19 were based on the New Coronavirus Pneumonia Prevention and Control Program (8th edition), published by the National Health Commission of China.^7^ The severity of COVID-19 infection was categorized as (1) mild, if there was no radiographic evidence of pneumonia; (2) moderate, if pneumonia was accompanied by fever and respiratory tract symptoms; (3) severe, if respiratory rate was ≥30/min, oxygen saturation was ≤93% when breathing ambient air, or PaO_2_/FiO_2_ ≤ 300 mm Hg (1 mm Hg = 0.133 kPa); or (4) critical, if there was respiratory failure requiring mechanical ventilation, shock, or organ failure requiring intensive care. According to the vaccination status of SARS-CoV-2, the 320 subjects were divided into the unvaccinated group (*n* = 24), Sinopharm vaccine group (*n* = 97), Sinovac vaccine group (*n* = 142), CanSino vaccine group (*n* = 48) and other vaccine group (including Anhui Zhifei, Lanzhou Bio vaccine, Changchun Bio vaccine, 9 cases in total, *n* = 9). Due to the small sample size (<10) of patients in the other vaccine group, no further analysis was conducted in this study. Therefore, the present study conducted an in-depth analysis of clinical data from 311 convalescent adult patients infected with Omicron variant.

### 2.2. Inclusion and exclusion criteria

Inclusion criteria: (1) Meeting the diagnostic criteria for SARS-CoV-2 negative result at admission [normal temperature for >3 days; obvious improvement in respiratory symptoms and acute exudative lesions on chest computed tomography images; twice successive negative results of SARS-CoV-2 RNA test (interval of at least 24 h)]; (2) Local patients admitted to the Tianjin area of China (non-imported patients); (3) Patient age ≥16 years. Exclusion criteria: (1) SARS-CoV-2 pathogen test was positive on admission; (2) Patients who cannot sign informed consent or had missing important case information; (3) Patients with allergies and immunodeficiency diseases; (4) Patients <16 years-old.

### 2.3. Collection of clinical and laboratory data

A retrospective analysis was performed on the clinical and laboratory test results of the 320 convalescent adult patients with Omicron variant in the Tianjin First Central Hospital on the seventh day after admission. General data [including gender, age, admission time, vaccination, underlying diseases (hypertension, diabetes, and coronary heart disease, etc.), clinical manifestations (including fever, cough, expectoration, chills, fatigue, headache/dizziness, sore throat/dry throat, nasal congestion/runny nose, muscle soreness, chest tightness, shortness of breath, and decreased smell/taste), the severity of illness, clinical symptoms on admission and nucleic acid re-positive during hospitalization] of patients in each group were reviewed and collected from the electronic medical records by HIS system of Tianjin First Central Hospital. Before discharge, clinical and laboratory test data of each patient were collected and compiled. Two researchers reviewed the data collection forms independently to verify data accuracy.

### 2.4. SARS-CoV-2 testing

Real time-polymerase-chain-reaction assays were performed following the WHO protocol to detect the two target genes, the open reading frame of 1ab (*ORF1ab*) and the nucleocapsid protein (*N*), in a Light-Cycler 480 real-time PCR system. The diagnostic reagents for SARS-CoV-2 infection were obtained from Guangzhou Daan Gene Co., Ltd, Guangzhou, China, and suspicious results were confirmed by using another reagent, Changsha SANSURE GENE Detection kit for 2019-nCoV, China. The Omicron variant was identified by the Centers for Disease Control and Prevention of Tianjin using gene sequencing. A positive result was defined as PCR Ct value <40. RNA diagnosis in patients during rehabilitation or post-discharge was defined as RP, otherwise, as non-RP (NRP).

### 2.5. Novel coronavirus serologic assays

Novel coronavirus-specific IgG and IgM were detected in serum samples using Chemiluminescence Microparticle Immuno Assay (CMIA) developed by Bioscience (Tianjin, China). Patient serum was sent to the laboratory for routine testing. The assay uses a recombinant antigen corresponding to the nucleocapsid protein (*N*) of the SARS-CoV-2 wild-type genome.

### 2.6. Study outcomes

The primary endpoint was COVID-19 severity. The disease severity was defined as asymptomatic, mild, moderate, severe, and critical according to the New Coronavirus Pneumonia Prevention and Control Program (8th edition), published by the National Health Commission of China (see text foot note 7). The secondary endpoints were re-positive (RP) results on nucleic acid tests, liver and kidney function, and inflammation levels during recovery. RP was defined as PCR Ct value <40 after two independent PCR-negative results at an interval of >24 h.

### 2.7. Statistical analysis

SPSS 22.0 (IBM Crop, Armonk, NY, USA) software was used for statistical analysis without filling in the missing data. Continuous variables are presented as median [interquartile range (IQR)] or mean (SD) and categorical variables as number and percentage [No. (%)]. Significant differences for continuous variables were compared using unpaired *t*-tests when the data were normally distributed, otherwise, Mann-Whitney U-test was used. Proportions for categorical variables were compared using the χ2 test or Fisher’s exact test. A binary logistic regression model was used to estimate the association between some potential factors and the severity of illness. *P*-value < 0.05 (two-sided) was considered statistically significant.

## 3. Results

### 3.1. Demographic and vaccination characteristics of convalescent adult patients infected with SARS-Cov-2 Omicron variant

**FIGURE 1:**
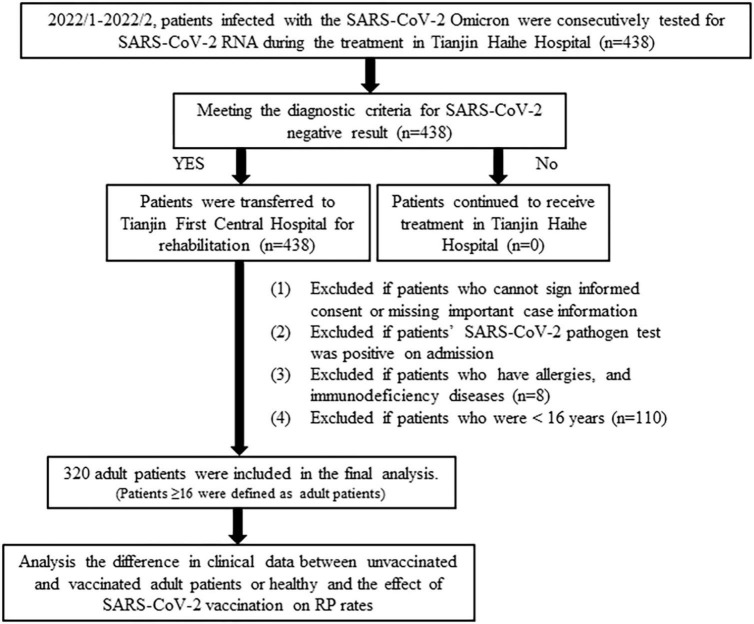
The flow chart of the critical of cases inclusion or exclusion.

The study flowchart showing the strategy of case inclusion or exclusion is illustrated in Figure 1. A total of 438 patients with Omicron infection were diagnosed in the Tianjin Haihe Hospital. The recovered patients meeting the diagnostic criteria for SARS-CoV-2 negative results were transferred to Tianjin First Central Hospital. Patients who could not sign the informed consent or had missing important case information or presented allergies and immunodeficiency diseases or were positive for SARS-CoV-2 pathogen test or age ≤16 years were excluded from further analysis. 320/438 (73.06%) adult patients recovered from COVID-19 met the inclusion criteria, including 24 (7.50%) unvaccinated adult patients, 97 (30.31%) adult patients vaccinated with Sinopharm vaccine (Sinopharm patients), 142 (44.38%) adult patients vaccinated with Sinovac vaccine (Sinovac patients), 48 (15.00%) adult patients vaccinated with CanSino vaccine (CanSino patients), and 9 (2.81%) adult patients vaccinated with other vaccines (including Anhui Zhifei vaccine, Lanzhou Bio vaccine, and Changchun Bio vaccine). Due to the extremely small sample size (<10) vaccinated with other vaccines, no further analysis was performed in this group of adult patients.

Table 1 shows the demographic data and vaccination information of adult patients on admission. The median (IQR) age of the 311 adult patients was 46 (34–58) years; 136 (43.73%) of them were male and 175 (56.27%) were women. There was no significant difference in the sex composition ratio between different groups (*P* > 0.05). The median age of unvaccinated patients, Sinopharm patients, Sinovac patients, and CanSino patients was 59 (33–75), 44 (34–58), 44 (34–58), and 48 (35–57) years, respectively. Obviously, the median age of unvaccinated patients was higher than that of the vaccinated patients. In addition, 67 (69.07%) Sinopharm and 87 (61.27%) Sinovac patients were vaccinated with an additional booster dose.

**TABLE 1:** Basic information of convalescent adult patients on admission.

	All patients (*n* = 311)	Unvaccination	Vaccination		
		Unvaccinated (*n* = 24)	Sinopharm (*n* = 97)	Sinovac (*n* = 142)	CanSino (*n* = 48)
**Gender**					
Male	136 (43.73)	6 (25.00)	49 (50.52)	62 (43.66)	19 (39.58)
Female	175 (56.27)	18 (75.00)	48 (49.48)	80 (56.34)	29 (60.42)
**Age**					
Median age (IQR), yr	46 (34–58)	59 (33–75)*^#△^	44 (34–58)^○^	44 (34–58)^○^	48 (35–57)^○^
16–39 years	125 (40.19)	7 (29.17)	44 (45.36)	59 (41.55)	15 (31.25)
40–59 years	120 (38.59)	5 (20.83)	36 (37.11)	52 (36.62)	27 (56.25)
≥60 years	66 (21.22)	12 (50.00)	17 (17.53)	31 (21.83)	6 (12.50)
**Dose**					
0 dose	24 (7.72)	24 (100.00)	–	–	–
1 dose	17 (5.47)	–	2 (2.06)	0 (0.00)	15 (31.25)
2 doses	116 (37.30)	–	28 (28.87)	55 (38.73)	33 (68.75)
3 doses (booster dose)	154 (49.52)	–	67 (69.07)	87 (61.27)	0 (0.00)

Data are presented as median (IQR) or No. (%). No. is the number of patients with available data.

^○^*P* < 0.05, compared with the unvaccinated group.

*P < 0.05, compared with the Sinopharm group.

^#^*P* < 0.05, compared with the Sinovac group.

^△^*P* < 0.05, compared with the CanSino group; *P* < 0.05 were statistically significant.

IQR, interquartile range; yr, year.

### 3.2. Clinical characteristics of convalescent adult patients with different vaccinations on admission

As shown in Table 2, the most common clinical symptoms were dry cough (38.91%) and fever (28.62%) in the 311 adult patients during convalescence, while some patients had abnormal taste (1.61%), abnormal sense of smell (1.29%), or diarrhea (1.29%). Strikingly, no significant difference was detected in the clinical symptoms between the different groups. Compared to unvaccinated patients (29.17%), Sinopharm (7.22%), Sinovac (9.15%), and CanSino patients (2.08%) had a lower proportion of coronary heart disease. Additionally, Sinopharm patients had a lower proportion of malignancy (1.03%) and chronic respiratory diseases (3.09%) compared to unvaccinated patients. Overall, the proportion of patients with comorbid underlying diseases in Sinopharm (56.70%), Sinovac (53.52%), and CanSino groups (47.92%) was significantly lower than that in the unvaccinated group (79.17%, *P* < 0.05).

**TABLE 2:** Clinical characteristics of convalescent adult patients in different groups.

	All patients (*n* = 311)	Unvaccination	Vaccination		
		Unvaccinated (*n* = 24)	Sinopharm (*n* = 97)	Sinovac (*n* = 142)	CanSino (*n* = 48)
Clinical symptoms	199 (63.99)	17 (70.83)	62 (63.92)	86 (60.56)	34 (70.83)
Fever	89 (28.62)	8 (33.33)	24 (24.74)	41 (28.87)	16 (33.33)
Stuffy nose	37 (11.90)	1 (4.17)	11 (11.34)	22 (15.49)	3 (6.25)
Runny nose	39 (12.54)	4 (16.67)	10 (10.31)	17 (11.97)	8 (16.67)
Dry cough	121 (38.91)	8 (33.33)	39 (40.21)	59 (41.55)	15 (31.25)
Weak	46 (14.79)	4 (16.67)	12 (12.37)	24 (16.90)	6 (12.50)
Sore throat	62 (19.94)	3 (12.50)	20 (20.62)	29 (20.42)	10 (20.83)
Abnormal taste	5 (1.61)	0 (0.00)	0 (0.00)	3 (2.11)	2 (4.17)
Abnormal smell	4 (1.29)	1 (4.17)	0 (0.00)	2 (1.41)	1 (2.08)
Inflammation of the Conjunctival mucosa	16 (5.14)	3 (12.50)	3 (3.09)	6 (4.23)	4 (8.33)
Diarrhea	4 (1.29)	0 (0.00)	1 (1.03)	2 (1.41)	1 (2.08)
Comorbid underlying conditions	173 (55.63)	19 (79.17)*^#△^	55 (56.70)^○^	76 (53.52)^○^	23 (47.92)^○^
Hypertension	91 (29.26)	9 (37.50)	26 (26.80)	45 (31.69)	11 (22.92)
Diabetes	48 (15.43)	5 (20.83)	17 (17.53)	23 (16.20)	3 (6.25)
Coronary heart disease	28 (9.00)	7 (29.17)*^#△^	7 (7.22)^○^	13 (9.15)^○^	1 (2.08)^○^
Chronic liver disease	8 (2.57)	1 (4.17)	1 (1.03)	6 (4.23)	0 (0.00)
Chronic kidney disease	9 (2.89)	1 (4.17)	3 (3.09)	5 (3.52)	0 (0.00)
Malignancy	10 (3.22)	3 (12.50)*	1 (1.03)^○^	6 (4.23)	0 (0.00)
Chronic respiratory diseases	14 (4.50)	4 (16.67)*	3 (3.09)^○^	6 (4.23)	1 (2.08)
Abnormal liver function	66 (21.22)	7 (29.17)	23 (23.71)	25 (17.61)	11 (22.92)

Data are presented as No. (%). No. is the number of patients with available data.

^○^*P* < 0.05, compared with the unvaccinated group.

*P < 0.05, compared with the Sinopharm group.

^#^*P* < 0.05, compared with the Sinovac group.

^△^*P* < 0.05, compared with the CanSino group; *P* < 0.05 were statistically significant.

### 3.3. Clinical classification of adult patients with different vaccinations at diagnosis

**FIGURE 2:**
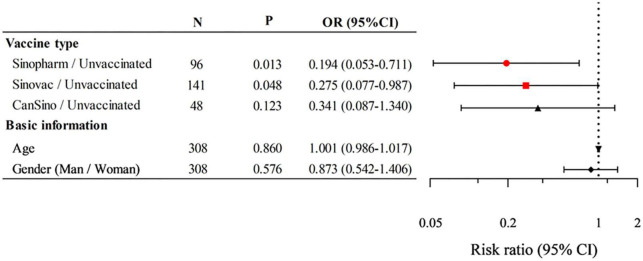
Logistic regression analysis of the risk and protective factors on the coronavirus disease 2019 (COVID-19) disease severity. *P* < 0.05 were statistically significant. *N*, total number of cases; OR, odds ratio; CI, confidence interval.

The effect of vaccines on the disease severity of adult patients infected with the Omicron variant was investigated. As shown in Table 3, compared to unvaccinated patients (12.50%), Sinopharm (44.33%), and Sinovac (35.92%) patients had a visible higher proportion of mild conditions (*P* < 0.05). Although no statistical difference was detected, CanSino patients (31.25%) had a higher proportion of mild conditions than unvaccinated patients (12.50%). Moreover, Sinopharm patients (54.64%) had a significantly lower proportion of moderate conditions compared to unvaccinated patients (83.33%, *P* < 0.05). Similarly, Sinovac (63.38%) and CanSino (68.75%) patients had a lower proportion of moderate conditions compared to unvaccinated patients (83.33%), albeit not significantly. The number of patients with severe conditions was too small for us to observe the effect of different vaccines on the rate of severe disease. In addition, the risk and protective factors of COVID-19 disease severity were analyzed by binary logistic regression (due to the extremely small sample size of asymptomatic and severe patients, regression analysis was not included.) (Figure 2). Binary logistic regression results suggested that the receipt of Sinopharm and Sinovac vaccines (as compared with unvaccinated patients) was an independent protective factor for relieving the severity of the Omicron variant infection (OR 0.194, 95% CI 0.053–0.711, *P* < 0.05); however, no significant correlation was established between gender, age and CanSino vaccine and disease severity. These results suggested that Sinopharm and Sinovac vaccines have better protective effects than CanSino vaccine in relieving the COVID-19 disease severity.

**TABLE 3:** Clinical classifications of adult patients in different groups.

	All patients (*n* = 311)	Unvaccination	Vaccination		
		Unvaccinated (*n* = 24)	Sinopharm (*n* = 97)	Sinovac (*n* = 142)	CanSino (*n* = 48)
Asymptomatic	1 (0.32)	0 (0.00)	1 (1.03)	0 (0.00)	0 (0.00)
Mild	112 (36.01)	3 (12.50)*^#^	43 (44.33)^○^	51 (35.92)^○^	15 (31.25)
Moderate	196 (63.02)	20 (83.33)*	53 (54.64)^○^	90 (63.38)	33 (68.75)
Severe	2 (0.64)	1 (4.17)	0 (0.00)	1 (0.70)	0 (0.00)

Data are presented as No. (%). No. is the number of patients with available data.

^○^P < 0.05, compared with the unvaccinated group.

*P < 0.05, compared with the Sinopharm group.

^#^P < 0.05, compared with the Sinovac group.

^△^P < 0.05, compared with the CanSino group; P < 0.05 were statistically significant.

### 3.4. Liver and kidney function-associated biomarkers of convalescent adult patients with different vaccinations

Although COVID-19 primarily affects the respiratory system, accumulating emerging evidence highlights the impact of this viral infection on other organs (Guan et al., 2020; Skok et al., 2021). To understand the effect of vaccines on the long-term health consequences of adult patients infected with Omicron, we collected and analyzed the clinical laboratory test data of the liver and kidney function-related indicators [alanine aminotransferase (ALT), aspartate aminotransferase (AST), total bilirubin (TB), creatinine (CREA), and blood urea nitrogen (BUN)] in convalescent adult patients. Table 4 summarizes the findings of laboratory examination related to liver and renal damage in convalescent adult patients. Interestingly, CanSino patients had significantly higher ALT levels [IQR 41.81 (24.79–85.43)] compared to unvaccinated patients [IQR 27.06 (15.67–42.67), *P* < 0.05], but no difference was observed between different groups in liver function-related indicators. Regarding the kidney function-related indicators, higher CREA levels were detected in Sinovac patients [IQR 60.57 (50.15–71.22)] than in unvaccinated patients [IQR 53.72 (43.52–65.49), *P* < 0.05]. Additionally, we did not find discernible differences in other indicators between convalescent patients in different groups. These results indicated that the three vaccines did not exert any protective effect on the liver and kidney functions of convalescent patients.

**TABLE 4:** The liver and kidney function-related indicators of convalescent adult patients in different groups.

	Liver function-related indicators			Kidney function-related indicators	
	ALT (U/L)	AST (U/L)	TB (μmol/L)	CREA (μmol/L)	BUN (mmol/L)
All patients (*n* = 311)	33.53 (20.48–66.38)	28.91 (23.15–38.93)	9.41 (6.96–11.93)	59.77 (49.35–70.88)	4.01 (3.33–4.89)
Unvaccinated (*n* = 24)	27.06 (15.67–42.67)^△^	26.47 (22.07–40.89)	9.36 (6.45–11.33)	53.72 (43.52–65.49)^#^	4.55 (3.36–6.30)
Sinopharm (*n* = 97)	33.55 (20.99–67.05)	30.87 (23.57–41.03)	9.27 (7.13–12.27)	60.72 (49.08–71.88)	4.05 (3.21–4.80)
Sinovac (*n* = 142)	33.51 (20.50–69.48)	28.82 (22.77–37.23)	9.20 (6.87–11.13)	60.57 (50.15–71.22)^○^	3.98 (3.36–4.81)
CanSino (*n* = 48)	41.81 (24.79–85.43)^○^	29.49 (24.01–38.54)	9.95 (7.59–13.70)	54.95 (48.84–73.23)	3.92 (3.47–4.94)

Data are presented as median (IQR). No. is the number of patients with available data.

^○^*P* < 0.05, compared with the unvaccinated group.

^#^*P* < 0.05, compared with the Sinovac group.

^△^*P* < 0.05, compared with the CanSino group; *P* < 0.05 were statistically significant.

ALT, alanine aminotransferase; AST, aspartate aminotransferase; TB, total bilirubin; CREA, creatinine; BUN, blood urea nitrogen.

### 3.5. Inflammatory and immune characteristics of convalescent adult patients with different vaccinations

The immune system plays an major role in antiviral infection and rehabilitation. To further study the effect of vaccines on the immune characteristics of convalescent adult patients infected with Omicron, we reviewed and analyzed the test data for inflammatory and immune-related indicators [interleukin-6 (IL-6), C-reactive protein (CRP), immunoglobulin G (IgG), immunoglobulin M (IgM), T lymphocytes (CD3^+^T, CD4^+^T, and CD8^+^T cell), and various blood cell counts] on day 7 after admission. As shown in Table 5, Sinopharm, Sinovac, and CanSino patients had significantly lower IL-6 and CRP levels compared to the unvaccinated patients (*P* < 0.05). Virus-specific IgG and IgM levels were notably lower in unvaccinated patients than in Sinopharm, Sinovac and CanSino patients. Moreover, Sinovac and CanSino patients had higher IgG levels than Sinopharm patients, and Sinovac patients had more higher IgM levels than CanSino patients. Additionally, Sinovac and CanSino patients had higher lymphocyte levels than unvaccinated patients (*P* < 0.05), while no visible differences were observed in WBCs, neutrophils, monocytes, eosinophils, and basophils between patients in different groups. Further analysis revealed that Sinovac patients had higher CD8^+^T cell levels and CanSino patients had higher CD3^+^T, CD4^+^T, and CD8^+^T cell levels compared to the unvaccinated patients, while no discernible differences were detected in T lymphocyte subsets between Sinopharm and unvaccinated patients. These results indicated that Sinovac and CanSino vaccines had better immune stimulation effects on the body’s defense against the virus than the Sinopharm vaccine in convalescent adult patients.

**TABLE 5:** The immune-related indicators of convalescent adult patients in different groups of patients.

	All patients (*n* = 311)	Unvaccination	Vaccination		
		Unvaccinated (*n* = 24)	Sinopharm (*n* = 97)	Sinovac (*n* = 142)	CanSino (*n* = 48)
IL-6 (pg/mL)	1.50 (1.50–2.81)	2.72 (1.50–6.81)*^#△^	1.50 (1.50–3.15)^○^	1.50 (1.50–2.24)^○^	1.50 (1.50–1.97)^○^
CRP (mg/L)	0.98 (0.40–1.99)	2.13 (0.43–5.36)*^#△^	0.96 (0.42–1.98)^○^	0.98 (0.44–1.81)^○^	0.79 (0.25–1.54)^○^
IgG (S/CO)	207.05 (179.34–236.74)	170.74 (1.94–185.48)*^#△^	192.55 (165.20–219.09)^○#△^	217.10 (188.32–241.17)^○^*	225.77 (203.55–248.16)^○^*
IgM (S/CO)	0.48 (0.26–0.87)	0.22 (0.17–0.49)*^#△^	0.41 (0.25–0.82)^○^	0.56 (0.33–1.09)^○△^	0.38 (0.22–0.81)^○#^
WBCs (10^9^/μL)	6.22 (5.23–7.39)	6.50 (4.79–7.84)	6.38 (5.18–7.49)	6.22 (5.48–7.23)	5.88 (5.01–7.89)
Neutrophils (10^9^/μL)	3.59 (2.85–4.37)	3.61 (2.64–4.76)	3.57 (2.80–4.46)	3.62 (2.92–4.07)	3.33 (2.79–4.83)
Monocytes (10^9^/μL)	0.42 (0.35–0.51)	0.49 (0.40–0.55)	0.42 (0.34–0.54)	0.41 (0.35–0.50)	0.43 (0.35–0.53)
Eosinophils (10^9^/μL)	0.13 (0.09–0.19)	0.14 (0.10–0.19)	0.11 (0.08–0.20)	0.13 (0.09–0.19)	0.13 (0.08–0.20)
Basophils (10^9^/μL)	0.03 (0.02–0.04)	0.03 (0.01–0.04)	0.02 (0.02–0.04)	0.03 (0.02–0.04)	0.02 (0.02–0.03)
Lymphocytes (10^9^/μL)	2.05 (1.61–2.42)	1.69 (1.24–2.54)^#△^	2.00 (1.56–2.41)	2.09 (1.75–2.40)^○^	2.07 (1.63–2.62)^○^
CD3^+^ T (count/μL)	67892.13 (37064.36–139377.88)	52046.63 (14400.53–77627.99)^○^	65805.89 (39097.01–125109.46)	70274.71 (36528.54–152185.69)	80881.90 (42175.90–151744.83)^○^
CD4^+^ T (count/μL)	38198.00 (18534.97–77080.73)	28339.47 (8471.35–49413.39)^○^	33531.67 (19889.02–71671.98)	40260.49 (18551.39–90744.26)	48035.72 (18823.59–89922.86)^○^
CD8^+^ T (count/μL)	23837.54 (11719.67–44116.00)	12420.81 (5319.62–26751.11)^#△^	22889.46 (11852.83–43335.03)	24152.62 (12165.85–44891.56)^○^	28575.31 (13363.10–52032.29)^○^

Data are presented as median (IQR). No. is the number of patients with available data.

^○^*P* < 0.05, compared with the unvaccinated group.

*P < 0.05, compared with the Sinopharm group.

^#^P<0.05, compared with the Sinovac group.

^△^*P* < 0.05, compared with the CanSino group; *P* < 0.05 were statistically significant.

CRP, C reaction protein; WBCs, white blood cells.

### 3.6. Comparison of RP rates of convalescent adult patients with different vaccinations

Several studies have described the RP results of the SARS-CoV-2 RNA test in recovered patients (An et al., 2020; Li et al., 2020, 2021; Qu et al., 2020). A total of 72 (23.15%) RP patients in this study, including 12 (16.67%) unvaccinated patients, 23 (31.94%) Sinopharm, 26 (36.11%) Sinovac, and 11 (15.28%) CanSino patients. However, the effect of vaccines on RP and NRP rate of convalescent adult patients was unclear. In order to monitor the health status of adult patients with Omicron variant infection during recovery, daily throat swab sampling and nucleic acid testing were conducted. As shown in Table 6, the RP rates of Sinopharm (23.71%), Sinovac (18.31%), and CanSino patients (22.92%) were significantly lower compared to unvaccinated patients (50.00%, *P* < 0.05), respectively. Accordingly, the vaccinated patients had notably higher NRP rates than unvaccinated patients. These results suggested that the three domestic vaccines have a protective effect on the prevention of RP in convalescent adult patients.

**TABLE 6:** The RP rate of convalescent adult patients in different groups.

	All patients (*n* = 311)	Unvaccination	Vaccination		
		Unvaccinated (*n* = 24)	Sinopharm (*n* = 97)	Sinovac (*n* = 142)	CanSino (*n* = 48)
RP (%)	72 (23.15)	12 (50.00)*^#△^	23 (23.71)^○^	26 (18.31)^○^	11 (22.92)^○^
NRP (%)	239 (76.85)	12 (50.00)*^#△^	74 (76.29)^○^	116 (81.69)^○^	37 (77.08)^○^

Data are presented as No. (%). No. is the number of patients with available data.

^○^*P* < 0.05, compared with the unvaccinated group.

*P < 0.05, compared with the Sinopharm group.

^#^*P* < 0.05, compared with the Sinovac group.

^△^*P* < 0.05, compared with the CanSino group; *P* < 0.05 were statistically significant.

RP, re-detectable positive patients; NRP, non-RP patients.

## 4. Discussion

Since December 2019, acute respiratory infectious diseases caused by SARS-CoV-2 have spread rapidly worldwide. Several variants of concern (VOCs) with different transmissibility and immune escape have emerged, and are capable of causing breakthrough infections in vaccinated individuals (Jamal et al., 2022; Lee et al., 2022; Zhang et al., 2022). The emergence of SARS-CoV-2 VOCs is associated with new waves of infections, sometimes across the entire world. The Omicron variant was first identified in South Africa in November 2021 and since then spread rapidly (WHO Update on Omicron. Nov 28, 2021).^8^ Now, it is the main variant circulating across many countries and regions, and the number of infections is rising continually (Karim and Karim, 2021; Gahukar and Khairnar, 2022).

Structure is essential for the pathogenicity of SARS-CoV-2. Omicron’s structural proteins include spike protein S, envelope protein M, matrix protein R, and nucleocapsid protein N. Among these, the surface spike protein S is composed of S1 and S2 subunits, which recognize the host cell receptor ACE2 through the receptor-binding domain (RBD), thereby infecting host cells. The S protein is the only viral protein that modifies the surface of virions and is the primary antigen target for natural infection and vaccine design (Giovanetti et al., 2021). Previous studies have reported that the Omicron variant has >30 mutations in the S protein, 15 of which are in the RBD, including K417N, S477N, T478K, E484A, N501Y, G339D, S371L, S373P, S375F, N440K, G446S, Q493R, G496S, Q498R, and Y505H, whereas the Delta variant has only two mutations (Pulliam et al., 2022). These 15 mutations are known to lead to increased transmissibility, higher viral binding affinity, and immune escape (Greaney et al., 2021; Harvey et al., 2021). Furthermore, the Omicron variant carries some of the same important mutations as the Delta and Alpha variants, which are also considered to be closely associated with its transmissibility and pathogenicity (Karim and Karim, 2021; Gahukar and Khairnar, 2022; Pulliam et al., 2022). Therefore, the Omicron variant through RBD of spike attaches to the ACE2 receptor and escapes the antibody response in variant complexes (Khan et al., 2022). Additionally, the SARS-CoV-2 genome encodes some accessory proteins that are essential for infection, including ORF3a, ORF3b, ORF6, ORF7a, ORF7b, ORF8, ORF9b, ORF9c, and ORF10 (Gordon et al., 2020; Zandi, 2022; Zandi et al., 2022). Notably, the main SARS-CoV-2 accessory proteins play significant roles in affecting immune escape and viral pathogenesis (Zandi et al., 2022), such as the regulation of cytokine synthesis by ORF9c or the inhibition of type I IFN activity by ORF3b, ORF6, ORF7a, ORF8, or ORF9b (Redondo et al., 2021; Hassan et al., 2022). Moreover, these key accessory proteins impair the host’s immune response through various mechanisms (Redondo et al., 2021; Hassan et al., 2022; Zandi et al., 2022). For instance, ORF3a, ORF7a, and ORF7b inhibit IFNα signaling, ORF8 suppresses IFNβ signaling, and ORF9c inhibits cytokine secretion (Xia et al., 2020). The above evidences suggested that structural and accessory proteins of SARS-CoV-2 play critical roles in accelerating the life cycle and evading host immune responses.

On January 8, 2022, the first non-imported Omicron infection case was reported in Tianjin, China (Tianjin Municipal Health Commission).^9^ As of March 15, 2022, the cumulative number of confirmed cases infected with SARS-Cov-2 Omicron variant in Tianjin city has reached 438, of whom 328 (74.89%) were adult patients. To further prevent and control the spread and transmission of the Omicron variant, mass vaccination programs have been carried out globally over the past 2 years. In mainland China, the most widely administered SARS-CoV-2 vaccine is the inactivated type (Lai et al., 2022), while adenovirus and recombinant protein vaccines were administered in fewer individuals. As of January 8, 2022, when the Omicron variant first emerged, Tianjin had reported that 30.446 million doses of SARS-CoV-2 vaccines administered, and 12.915 million people had been vaccinated. The proportion of residents in Tianjin who had been vaccinated to different degrees was 93.14% (Tianjin Municipal Health Commission).^10^ Nevertheless, such high vaccination rates have also not succeeded in eliminating the infection of the Omicron variant, and the effect of SARS-CoV-2 vaccines on the clinical characteristics of adult patients needs to be explored further. In this study, we analyzed and compared the clinical features and laboratory data between different vaccine groups and evaluated the effect of three domestic SARS-CoV-2 vaccines on the clinical characteristics, liver and kidney damage indicators, immune and inflammation indicators, and RP rates of convalescent adult patients. We observed that the age of unvaccinated patients was significantly higher than that of each vaccine group. A possible explanation may be that the elderly individuals had more comorbid underlying diseases and therefore did not meet the vaccination conditions, and some had less willingness to be vaccinated. Due to weak awareness of seeking medical care, weaker immunity and the lower rate of vaccinations, older people are more likely to be affected by Omicron variant infection than younger individuals.

Consistent with the previous findings, the clinical classification of adult patients with Omicron infection included in this study at the time of diagnosis was mainly mild and moderate. Sinopharm, Sinovac, and CanSino patients had a higher proportion of mild conditions compared to unvaccinated patients, while unvaccinated patients had a higher proportion of moderate and severe conditions compared to each vaccine group (Not all of those were significantly different). In addition, binary logistic regression result showed that Sinopharm and Sinovac vaccines were independent protective factors for relieving the severity of adult patients with Omicron infection. These results indicated that Sinopharm and Sinovac vaccines have better protective effects than CanSino vaccine in relieving the disease severity of adult patients infected with Omicron, although the CanSino vaccine also had a degree of protection against adult patients.

To date, most studies of the Omicron variant have focused on adult patients in the acute infectious phase, while studies on convalescent adult patients infected with Omicron are yet limited. Although SARS-CoV-2 primarily affects the respiratory system, several studies highlight the impact of this viral infection on other organs (Guan et al., 2020; Xu L. et al., 2020; Zhang C. et al., 2020; Skok et al., 2021; Jansen et al., 2022). Deng et al. (2022) reported that patients with SARS-CoV-2 infection might develop liver damage. To understand the effect of SARS-CoV-2 vaccines on long-term health consequences of adult patients infected with Omicron, we collected and analyzed the liver and kidney function-related data from convalescent adult patients. Surprisingly, we did not observe a protective effect of the vaccine on the liver and kidney function biomarkers in convalescent adult patients. Conversely, compared to unvaccinated patients, CanSino patients had higher ALT levels and Sinovac patients had higher CREA levels, although the cause underlying this phenomenon is yet to be elucidated. It could be attributed to the fact that some vaccinated patients with underlying liver and kidney disease had higher ALT and CREA levels, which masked the impact of the vaccine on the liver and kidney function indicators. On the other hand, the enrolled patients were in recovery and hence, the effect of Omicron on their liver and kidney function was fading.

Chen R. et al. (2020) showed that progressively elevated IL-6 levels constitute the risk factor for fatal outcomes with COVID-19, such that IL-6 may be a potential therapeutic target for patients with severe/critical COVID-19. In addition, CRP is also positively correlated with the severity of COVID-19 and associated with the clinical outcomes of the disease (Chen R. et al., 2020). In the present study, we found that IL-6 and CRP levels in vaccinated patients were significantly lower than in unvaccinated patients during recovery, suggesting that the three domestic vaccines have a significant protective effect on the resolution of inflammation in convalescent adult patients. Moreover, vaccinated patients had notably higher virus-specific IgG and IgM levels than unvaccinated patients, which indicated that the three domestic vaccines promoted adult patients to obtain a strong specific immune response-ability. Interestingly, we observed that Sinovac and CanSino patients had higher IgG levels than Sinopharm patients. This result suggested that Sinovac and CanSino vaccines have a better effect on promoting the production of SARS-CoV-2-specific IgG than Sinopharm vaccine in vaccinated adult patients.

Immune cells play a decisive role in maintaining immune homeostasis and inflammatory response throughout the body. Understanding the effect of vaccines on immune cell levels would provide an effective strategy for the prevention and treatment of COVID-19. Lymphopenia is an effective and reliable indicator of the severity and hospitalization in COVID-19 patients (Tan et al., 2020). Additionally, most COVID-19 vaccines have remained effective in preventing severe COVID-19, hospitalization, and death, for all previous variants, because the efficacy might be more dependent on T cell immune responses than antibodies. In this study, compared with the unvaccinated patients, Sinovac and CanSino patients had higher lymphocyte levels than the unvaccinated patients. Studies in adult patients have deduced the importance of CD4^+^ T cells in controlling and fine-tuning the pathogenesis and outcomes of SARS-CoV and Middle East respiratory syndrome CoV infection (Chen G. et al., 2020). Another study indicated that SARS-CoV-2 vaccination specifically affected the cell-mediated immunity profile of convalescent pediatric patients, especially CD3^+^ T and CD8^+^ T cells (Wang et al., 2022). Interestingly, CD4^+^ T and CD8^+^ T cells levels were obviously higher in CanSino patients than in unvaccinated patients, and Sinovac patients have significantly higher CD8^+^ T cell levels compared to unvaccinated patients. These results suggested that Sinovac and CanSino vaccines have notable effects on T lymphocyte levels in convalescent adult patients, which might promote the recovery of adult patients infected with Omicron variants. Consistent with the previous studies (Wang et al., 2022), we observed that the Sinopharm, Sinovac, and CanSino vaccines have significant protective effects on the prevention of RP in convalescent adult patients. Taken together, we inferred that although vaccination does not eliminate infection of the Omicron variant, it has a significant effect on the body’s defense against the virus in adults.

To the best of our knowledge, this is the first study to comparatively analyze the differences in clinical and immunological features of convalescent adult patients receiving different SARS-CoV-2 vaccines after Omicron infection. Briefly, this study showed that although the three domestic SARS-CoV-2 vaccines could not resist the invasion of the Omicron variant, they exerted a protective role in relieving the severity of COVID-19 disease and reducing the level of inflammation and the RP rate of convalescent adult patients, while increasing body’s defense against the virus in convalescent adult patients. The current findings support the national policy to promote vaccination, and while each vaccine might have different advantages from different perspectives, they are all beneficial to adult patients. In addition, our findings provided novel insights into realizing the effect of SARS-CoV-2 vaccines on the long-term health of adult patients and a reference for the prevention and treatment measures for adults infected with Omicron variants.

Over the past 3 years, the rapid development and deployment of effective SARS-CoV-2 vaccines have greatly reduced the risk of severe illness and death associated with COVID-19, while vaccination does not seem to have succeeded in controlling and preventing the pandemic. Although most of the adult patients have been vaccinated prior to Omicron infection in this study, they are still infected with the virus, showing that vaccination cannot completely resist the invasion of the Omicron variants. Additionally, due to its ability to rapidly evolve and mutate, the SARS-CoV-2 virus may never be eradicated, thereby there are many important new topics to work on if we need to live with SARS-CoV-2 virus for a long time.

Nevertheless, the present study has several limitations. First, this was a retrospective and single-center study, and additional studies with large multi-center samples are needed. A small sample size of patients may lead to bias in experimental results, especially only 24 patients in the unvaccinated group. Second, due to the lack of laboratory data on the serum-specific neutralizing antibodies against Omicron variants in patients, the clinical effect of vaccines against the Omicron variant cannot be evaluated accurately. Thus, a comprehensive supplement is required in the following studies. Third, since all the clinical data were collected retrospectively, some individuals had incomplete laboratory-related information.

## Data availability statement

The raw data supporting the conclusions of this article will be made available by the authors, without undue reservation.

## Ethics statement

The studies involving human participants were reviewed and approved by the Ethics Committee of Tianjin First Central Hospital. The patients/participants provided their written informed consent to participate in this study. Written informed consent was obtained from the individual(s) for the publication of any potentially identifiable images or data included in this article.

## Author contributions

HM and CZ: study concept and design. JW: design and drafting of the manuscript. HD: critical review of the manuscript and data analysis. JZ, TL, and MW: data collection, analysis, and interpretation. All authors contributed to the article and approved the submitted version.

## Footnotes

1
https://www.who.int/news/item/26-11-2021-classification-of-omicron-(b.1.1.529)-sars-cov-2-variant-of-concern

2
https://covid19.who.int/

3
http://www.nhc.gov.cn/xcs/yqfkdt/202201/1c1a23e9e172481da9a6b087c3eaa8fe.shtml

4
https://ourworldindata.org/covid-vaccinations

5
http://www.nhc.gov.cn/xcs/s3574/202207/f920442a8a4845e7833ca8d7e7cd876e.shtml

6
http://health.people.com.cn/n1/2022/0315/c14739-32375333.html

7
http://www.gov.cn/zhengce/zhengceku/2021-04/15/content_5599795.htm

8
https://www.who.int/news/item/28-11-2021-update-on-Omicron

9
https://wsjk.tj.gov.cn/ZTZL1/ZTZL750/YQFKZL9424/FKDT1207/202201/t20220109_5774785.html

10
https://wsjk.tj.gov.cn/ZTZL1/ZTZL750/YQFKZL9424/FKDT1207/202201/t20220102_5769364.html

## References

[B1] An J. Liao X. Xiao T. Qian S. Yuan J. Ye H. (2020). Clinical characteristics of recovered COVID-19 patients with re-detectable positive RNA test. *Ann. Transl. Med.* 8:1084. 10.21037/atm-20-5602 33145303 PMC7575971

[B2] CDC COVID-19 Response Team (2021). SARS-CoV-2 B.1.1.529 (omicron) variant—united states, december 1-8, 2021. *MMWR Morb. Mortal. Wkly. Rep.* 70 1731–1734. 10.15585/mmwr.mm7050e1 34914670 PMC8675659

[B3] Chen G. Wu D. Guo W. Cao Y. Huang D. Wang H. (2020). Clinical and immunological features of severe and moderate coronavirus disease 2019. *J. Clin. Invest.* 130 2620–2629. 10.1172/jci137244 32217835 PMC7190990

[B4] Chen R. Sang L. Jiang M. Yang Z. Jia N. Fu W. (2020). Longitudinal hematologic and immunologic variations associated with the progression of COVID-19 patients in China. *J. Allergy Clin. Immunol.* 146 89–100. 10.1016/j.jaci.2020.05.003 32407836 PMC7212968

[B5] Chen Y. Chen L. Deng Q. Zhang G. Wu K. Ni L. (2020). The presence of SARS-CoV-2 RNA in the feces of COVID-19 patients. *J. Med. Virol.* 92 833–840. 10.1002/jmv.25825 32243607

[B6] Chia W. Zhu F. Ong S. Young B. Fong S. Le Bert N. (2021). Dynamics of SARS-CoV-2 neutralising antibody responses and duration of immunity: A longitudinal study. *Lancet Microbe* 2 e240–e249. 10.1016/s2666-5247(21)00025-2 33778792 PMC7987301

[B7] Deng H. Mai Y. Liu H. Guan J. (2022). Clinical characteristics of liver injury in SARS-CoV-2 Omicron variant- and Omicron subvariant-infected patients. *Ann. Hepatol.* 28:100763. 10.1016/j.aohep.2022.100763 36182032 PMC9515007

[B8] Fiolet T. Kherabi Y. MacDonald C. J. Ghosn J. Peiffer-Smadja N. (2022). Comparing COVID-19 vaccines for their characteristics, efficacy and effectiveness against SARS-CoV-2 and variants of concern: A narrative review. *Clin. Microbiol. Infect.* 28 202–221. 10.1016/j.cmi.2021.10.005 34715347 PMC8548286

[B9] Gahukar A. M. Khairnar K. (2022). Omicron variant of SARS-CoV-2: A pandemic of global concern warrants a cautious approach. *J. Med. Virol.* 95:e28211. 10.1002/jmv.28211 36224097 PMC9874656

[B10] Giovanetti M. Benedetti F. Campisi G. Ciccozzi A. Fabris S. Ceccarelli G. (2021). Evolution patterns of SARS-CoV-2: Snapshot on its genome variants. *Biochem. Biophys. Res. Commun.* 538 88–91. 10.1016/j.bbrc.2020.10.102 33199021 PMC7836704

[B11] Goh G. K. Dunker A. K. Uversky V. (2013). Prediction of intrinsic disorder in MERS-CoV/HCoV-EMC supports a high oral-fecal transmission. *PLoS Curr.* 5:aa6498b. 10.1371/currents.outbreaks.22254b58675cdebc256dbe3c5aa6498b 24270586 PMC3828228

[B12] Gordon D. Jang G. Bouhaddou M. Xu J. Obernier K. White K. (2020). A SARS-CoV-2 protein interaction map reveals targets for drug repurposing. *Nature* 583 459–468. 10.1038/s41586-020-2286-9 32353859 PMC7431030

[B13] Greaney A. Starr T. Gilchuk P. Zost S. Binshtein E. Loes A. (2021). Complete mapping of mutations to the SARS-CoV-2 spike receptor-binding domain that escape antibody recognition. *Cell Host Microbe* 29 44–57.e9. 10.1016/j.chom.2020.11.007 33259788 PMC7676316

[B14] Guan W. J. Ni Z. Y. Hu Y. Liang W. H. Ou C. Q. He J. X. (2020). Clinical characteristics of coronavirus disease 2019 in China. *N. Engl. J. Med.* 382 1708–1720. 10.1056/NEJMoa2002032 32109013 PMC7092819

[B15] Harvey W. Carabelli A. Jackson B. Gupta R. Thomson E. Harrison E. (2021). SARS-CoV-2 variants, spike mutations and immune escape. *Nat. Rev. Microbiol.* 19 409–424. 10.1038/s41579-021-00573-0 34075212 PMC8167834

[B16] Hassan S. Choudhury P. Dayhoff G. II. Aljabali A. Uhal B. Lundstrom K. (2022). The importance of accessory protein variants in the pathogenicity of SARS-CoV-2. *Arch. Biochem. Biophys.* 717:109124. 10.1016/j.abb.2022.109124 35085577 PMC8785432

[B17] He P. Liu B. Gao X. Yan Q. Pei R. Sun J. (2022). SARS-CoV-2 delta and omicron variants evade population antibody response by mutations in a single spike epitope. *Nat. Microbiol.* 7 1635–1649. 10.1038/s41564-022-01235-4 36151403 PMC9519457

[B18] Heneghan C. J. Spencer E. A. Brassey J. Plüddemann A. Onakpoya I. J. Evans D. H. (2021). SARS-CoV-2 and the role of orofecal transmission: A systematic review. *F1000Res* 10:231. 10.12688/f1000research.51592.2 35035883 PMC8749895

[B19] Jamal Z. Haider M. Ikram A. Salman M. Rana M. Rehman Z. (2022). Breakthrough cases of omicron and delta variants of SARS-CoV-2 during the fifth wave in Pakistan. *Front. Public Health* 10:987452. 10.3389/fpubh.2022.987452 36249252 PMC9557048

[B20] Jansen J. Reimer K. Nagai J. Varghese F. Overheul G. de Beer M. (2022). SARS-CoV-2 infects the human kidney and drives fibrosis in kidney organoids. *Cell Stem Cell* 29 217–231.e8. 10.1016/j.stem.2021.12.010 35032430 PMC8709832

[B21] Kannan S. Shaik Syed Ali P. Sheeza A. (2021). Omicron (B.1.1.529)—variant of concern - molecular profile and epidemiology: A mini review. *Eur. Rev. Med. Pharmacol. Sci.* 25 8019–8022. 10.26355/eurrev_202112_27653 34982466

[B22] Karim S. S. A. Karim Q. A. (2021). Omicron SARS-CoV-2 variant: A new chapter in the COVID-19 pandemic. *Lancet* 398 2126–2128. 10.1016/s0140-6736(21)02758-6 34871545 PMC8640673

[B23] Khan A. Waris H. Rafique M. Suleman M. Mohammad A. Ali S. (2022). The omicron (B.1.1.529) variant of SARS-CoV-2 binds to the hACE2 receptor more strongly and escapes the antibody response: Insights from structural and simulation data. *Int. J. Biol. Macromol.* 200 438–448. 10.1016/j.ijbiomac.2022.01.059 35063482 PMC8767976

[B24] Lai D. Xue J. He P. Jiang H. Li Y. Ma M. (2022). Longitudinal neutralization activities on authentic Omicron variant provided by three doses of BBIBP-CorV vaccination during one year. *Proteomics* 23:e2200306. 10.1002/pmic.202200306 36205637 PMC9874883

[B25] Lee J. Park S. Kim J. Lim S. Chang E. Bae S. (2022). No correlation of neutralizing antibody titers against the omicron variant after a booster dose of COVID-19 vaccines with subsequent breakthrough Omicron infections among healthcare workers. *J. Infect.* 85 e177–e180. 10.1016/j.jinf.2022.10.007 36223860 PMC9548087

[B26] Li Q. Shuai L. Tan L. Song L. Ou C. Song X. (2021). Clinical characteristics and prediction analysis of the recovered COVID-19 patients with re-detectable positive RNA test. *Am. J. Transl. Res.* 13 14157–14167. 35035761 PMC8748099

[B27] Li Y. Hu Y. Yu Y. Zhang X. Li B. Wu J. (2020). Positive result of Sars-Cov-2 in faeces and sputum from discharged patients with COVID-19 in Yiwu, China. *J. Med. Virol.* 92 1938–1947. 10.1002/jmv.25905 32311109 PMC7264799

[B28] Lopez Bernal J. Andrews N. Gower C. Robertson C. Stowe J. Tessier E. (2021). Effectiveness of the Pfizer-BioNTech and Oxford-AstraZeneca vaccines on COVID-19 related symptoms, hospital admissions, and mortality in older adults in England: Test negative case-control study. *BMJ* 373:n1088. 10.1136/bmj.n1088 33985964 PMC8116636

[B29] Lu R. Zhao X. Li J. Niu P. Yang B. Wu H. (2020). Genomic characterisation and epidemiology of 2019 novel coronavirus: Implications for virus origins and receptor binding. *Lancet* 395 565–574. 10.1016/s0140-6736(20)30251-8 32007145 PMC7159086

[B30] Malik J. Ahmed S. Mir A. Shinde M. Bender O. Alshammari F. (2022). The SARS-CoV-2 mutations versus vaccine effectiveness: New opportunities to new challenges. *J. Infect. Public Health* 15 228–240. 10.1016/j.jiph.2021.12.014 35042059 PMC8730674

[B31] McNamara L. Wiegand R. Burke R. Sharma A. Sheppard M. Adjemian J. (2022). Estimating the early impact of the US COVID-19 vaccination programme on COVID-19 cases, emergency department visits, hospital admissions, and deaths among adults aged 65 years and older: An ecological analysis of national surveillance data. *Lancet* 399 152–160. 10.1016/s0140-6736(21)02226-1 34741818 PMC8565933

[B32] Priyanka Choudhary O. Singh I. Patra G. (2020). Aerosol transmission of SARS-CoV-2: The unresolved paradox. *Travel Med. Infect. Dis.* 37:101869. 10.1016/j.tmaid.2020.101869 32891726 PMC7471761

[B33] Pulliam J. van Schalkwyk C. Govender N. von Gottberg A. Cohen C. Groome M. (2022). Increased risk of SARS-CoV-2 reinfection associated with emergence of omicron in South Africa. *Science* 376:eabn4947. 10.1126/science.abn4947 35289632 PMC8995029

[B34] Qu Y. M. Kang E. M. Cong H. Y. (2020). Positive result of Sars-Cov-2 in sputum from a cured patient with COVID-19. *Travel Med. Infect. Dis.* 34:101619. 10.1016/j.tmaid.2020.101619 32160971 PMC7129439

[B35] Rana R. Kant R. Huirem R. S. Bohra D. Ganguly N. K. (2022). Omicron variant: Current insights and future directions. *Microbiol. Res.* 265:127204. 10.1016/j.micres.2022.127204 36152612 PMC9482093

[B36] Redondo N. Zaldívar-López S. Garrido J. J. Montoya M. (2021). SARS-CoV-2 accessory proteins in viral pathogenesis: Knowns and unknowns. *Front. Immunol.* 12:708264. 10.3389/fimmu.2021.708264 34305949 PMC8293742

[B37] Sabino E. Buss L. Carvalho M. Prete C. Jr. Crispim M. Fraiji N. (2021). Resurgence of COVID-19 in Manaus, Brazil, despite high seroprevalence. *Lancet* 397 452–455. 10.1016/s0140-6736(21)00183-5 33515491 PMC7906746

[B38] Schmidt F. Weisblum Y. Rutkowska M. Poston D. DaSilva J. Zhang F. (2021). High genetic barrier to SARS-CoV-2 polyclonal neutralizing antibody escape. *Nature* 600 512–516. 10.1038/s41586-021-04005-0 34544114 PMC9241107

[B39] Skok K. Stelzl E. Trauner M. Kessler H. H. Lax S. F. (2021). Post-mortem viral dynamics and tropism in COVID-19 patients in correlation with organ damage. *Virchows Arch.* 478 343–353. 10.1007/s00428-020-02903-8 32815036 PMC7438212

[B40] Štefan M. Dlouhı P. Bezdíèková L. (2021). [Vaccination against COVID-19]. *Klin. Mikrobiol. Infekc. Lek.* 27 49–60. 34648642

[B41] Sun Y. Wang B. Zhang G. Zhang X. Wang C. Wang D. (2022). [Clinical characteristics of convalescent children infected with SARS-CoV-2 omicron variant in Tianjin]. *Zhonghua Er Ke Za Zhi* 60 1054–1058. 10.3760/cma.j.cn112140-20220711-00631 36207853

[B42] Tan L. Wang Q. Zhang D. Ding J. Huang Q. Tang Y. (2020). Lymphopenia predicts disease severity of COVID-19: A descriptive and predictive study. *Signal Transduct. Target. Ther.* 5:33. 10.1038/s41392-020-0148-4 32296069 PMC7100419

[B43] Tang S. Mao Y. Jones R. Tan Q. Ji J. Li N. (2020). Aerosol transmission of SARS-CoV-2? Evidence, prevention and control. *Environ. Int.* 144:106039. 10.1016/j.envint.2020.106039 32822927 PMC7413047

[B44] Tian D. Sun Y. Xu H. Ye Q. (2022). The emergence and epidemic characteristics of the highly mutated SARS-CoV-2 omicron variant. *J. Med. Virol.* 94 2376–2383. 10.1002/jmv.27643 35118687 PMC9015498

[B45] Wang J. Li T. Zhou C. Zhao J. Wang M. Wang Y. (2022). Clinical and immunological features of convalescent pediatric patients infected with the SARS-CoV-2 omicron variant in Tianjin, China. *Virol. Sin.* 37 850–859. 10.1016/j.virs.2022.10.009 36328182 PMC9621613

[B46] Wang K. Long Q. Deng H. Hu J. Gao Q. Zhang G. (2021). Longitudinal dynamics of the neutralizing antibody response to severe acute respiratory syndrome coronavirus 2 (SARS-CoV-2) infection. *Clin. Infect. Dis.* 73 e531–e539. 10.1093/cid/ciaa1143 32745196 PMC7454328

[B47] Xia H. Cao Z. Xie X. Zhang X. Chen J. Wang H. (2020). Evasion of type i interferon by SARS-CoV-2. *Cell Rep.* 33:108234. 10.1016/j.celrep.2020.108234 32979938 PMC7501843

[B48] Xu L. Liu J. Lu M. Yang D. Zheng X. (2020). Liver injury during highly pathogenic human coronavirus infections. *Liver Int.* 40 998–1004. 10.1111/liv.14435 32170806 PMC7228361

[B49] Xu Y. Li X. Zhu B. Liang H. Fang C. Gong Y. (2020). Characteristics of pediatric SARS-CoV-2 infection and potential evidence for persistent fecal viral shedding. *Nat. Med.* 26 502–505. 10.1038/s41591-020-0817-4 32284613 PMC7095102

[B50] Zandi M. (2022). ORF9c and ORF10 as accessory proteins of SARS-CoV-2 in immune evasion. *Nat. Rev. Immunol.* 22:331. 10.1038/s41577-022-00715-2 35361899 PMC8970066

[B51] Zandi M. Shafaati M. Kalantar-Neyestanaki D. Pourghadamyari H. Fani M. Soltani S. (2022). The role of SARS-CoV-2 accessory proteins in immune evasion. *Biomed. Pharmacother.* 156:113889. 10.1016/j.biopha.2022.113889 36265309 PMC9574935

[B52] Zhang C. Shi L. Wang F. S. (2020). Liver injury in COVID-19: Management and challenges. *Lancet Gastroenterol. Hepatol.* 5 428–430. 10.1016/s2468-1253(20)30057-1 32145190 PMC7129165

[B53] Zhang X. Wu S. Wu B. Yang Q. Chen A. Li Y. (2020). SARS-CoV-2 omicron strain exhibits potent capabilities for immune evasion and viral entrance. *Signal Transduct. Target. Ther.* 6:430. 10.1038/s41392-021-00852-5 34921135 PMC8678971

[B54] Zhang Y. Han S. Guo X. Yao M. Zhao L. Sun W. (2022). Breakthrough Infection shapes humoral immunity against SARS-CoV-2 omicron variant. *J. Infect.* 86 e40–e42. 10.1016/j.jinf.2022.10.021 36273640 PMC9584761

[B55] Zhang Y. Zeng G. Pan H. Li C. Hu Y. Chu K. (2021). Safety, tolerability, and immunogenicity of an inactivated SARS-CoV-2 vaccine in healthy adults aged 18-59 years: A randomised, double-blind, placebo-controlled, phase 1/2 clinical trial. *Lancet Infect. Dis.* 21 181–192. 10.1016/s1473-3099(20)30843-4 33217362 PMC7832443

